# Supplementary material for: In Vivo Evaluation of ECP Peptide Analogues for the Treatment of Acinetobacter baumannii Infection
Source: Biomedicines. 2022 Feb 5;10(2):386. doi: 10.3390/biomedicines10020386 (PMC8962335; doi:10.3390/biomedicines10020386)
Supplement: Supplementary file 1 [file biomedicines-10-00386-s001.zip › biomedicines-1553351-supplementary.pdf]

biomedicines

Supplementary Materials

In Vivo Evaluation of ECP Peptide Analogues for the Treatment of *Acinetobacter baumannii* Infection

Jiarui Li [1], Guillem Prats-Ejarque [1], Marc Torrent [1], David Andreu [2], Klaus Brandenburg [3], Pablo Fernández-Millán [1] and Ester Boix [1,*]

[1] Department of Biochemistry and Molecular Biology, Faculty of Biosciences, UAB, Cerdanyola del Vallès, Spain; jiarui.li@e-campus.uab.cat (JL), Guillem.Prats.Ejarque@uab.cat (GPE), Marc.Torrent@uab.cat (MT), Pablo.fernandez@uab.cat (PFM), Ester.Boix@uab.cat (EB)
[2] Department of Experimental and Health Sciences, Universitat Pompeu Fabra, Barcelona Biomedical Research Park, Barcelona, Spain; david.andreu@upf.edu
[3] Brandenburg Antiinfektiva GmbH, c/o Forschungszentrum Borstel, Borstel, Germany; kbranden@gmx.de
* Correspondence: Ester.Boix@uab.cat

Table S1. Sample sizes and administration times in each group for the main toxicity study.

Group	Dose (mg/kg)	Sample size (number of mice)	Day 0 A.M.	Day 0 P.M.	Day 1 A.M.	Day 1 P.M.	Day 2 A.M.	Day 2 P.M.
Vehicle (HBS)		3	✓	✓	✓	✓	✓	✓
ECPep-D	20	6	✓		✓			
	15	6	✓	✓		✓		✓
	7.5	1	✓		✓		✓	
ECPep-2D-Orn	25	6	✓	✓	✓		✓	
	20	6	✓	✓	✓		✓	
	10	2	✓		✓		✓	
	7.5	2	✓	✓	✓	✓	✓	✓

✓means administration at the corresponding time. A.M. means administration in the morning (around 9:00) and P.M. means administration in the afternoon (around 20:00). Owing to uncertainty of clinical reaction of the mice, the time planned to perform the following administration was decided based on the clinical symptoms observed after the previous administration dose.

Table S2. Assessment of general health status.

Clinical assessment	Score definition*
Body weight	0: body weight gain<br>1: no body weight gain/body weight loss <10% of the initial body weight<br>2: body weight loss between 10-20%<br>3: body weight loss >20%
Motor activity	0: normal<br>1: decreased motor activity, lameness, ataxia<br>2: need to be forced to stand up, partial paralysis<br>3: prostration, complete paralysis, dose no stand up when forced
General appearance	0: normal<br>1: less grooming than normal, mild piloerection<br>2: moderate piloerection and lesions in the coat, hunched posture<br>3: severe piloerection and lesions in the coat (ulcerative dermatitis)
Behaviour	0: normal/social behaviour<br>1: excitation/depressed<br>2: exacerbated reactions to external stimuli/no reaction to external stimuli<br>3: aggressiveness/stupor
Secretions	0: no secretion<br>1: mild secretion<br>2: severe secretion<br>3: haemorrhage/purulent secretion
Hydronation status	0: normal<br>1: long skin-tent duration<br>3: long skin-tent duration + enophthalmos
Breathing	0: normal<br>1: mild dyspnoea or tachypnoea<br>3: moderate dyspnoea or tachypnoea + prostration
Hypovolemia signs	0: mucous rose-coloured<br>1: mucous paleness<br>2: moderate mucous paleness and dry<br>3: moderate mucous paleness and dry + tachypnoea
Urination and defecation	1: feces and/or urine in very small quantities or slightly dark/orange or another colour appearance<br>2: feces and/or dark/pink urine excretion indication blood. appreciation of dilated bladder and/or hardened abdomen<br>3: dark feces and dark pink urine excretion indication blood. appreciation of dilated bladder and hardened abdomen, stained perineal area. completely liquid feces

* Sum of the score: 0-2: No alterations. Procedure will be continued; 3-6: Monitoring will be intensified. ≥ 7: Euthanasia will be considered.

Table S3. Cytotoxicity of ECP-derived peptides and colistin.

	**IC_{50}# (HEK293T)**	
	**48h**	
	**μM**	**μg/mL**
**ECPep-D**	63.60 ± 5.88	239.00 ± 22.10
**ECPep-2D-Orn**	>100	>350.52
**Colistin**	>300	>380.26

#IC_{50} is the cytotoxic concentration of the agents to cause death to 50% of viable cells. Each assay was done in triplicate by the MTT assay and normalized by no-treated control. Values are presented by Mean ± SE. N.D. indicates that no reduction of cell viability was detected at the highest concentration.

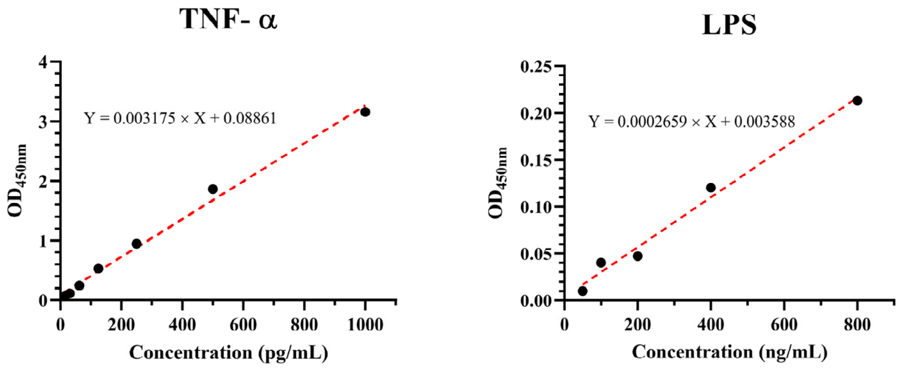

Figure S1. Standard curve of TNF-α and LPS tested by ELISA. The curve and the equation of the TNF-α and LPS concentration relative to OD_{450} obtained from the curve fit by linear regression.

a) ECPep-L

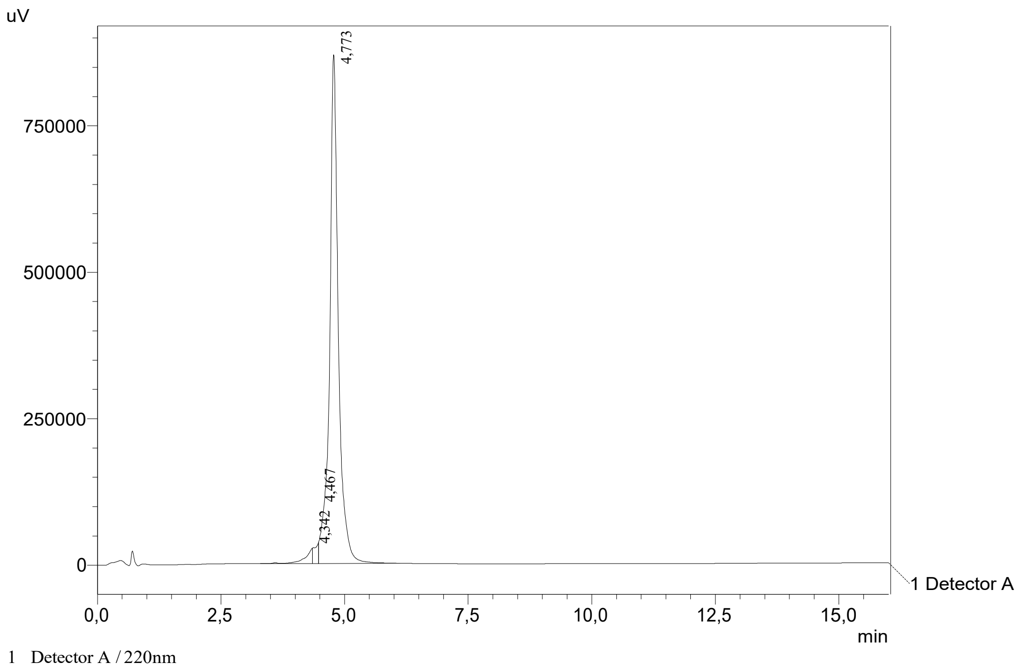

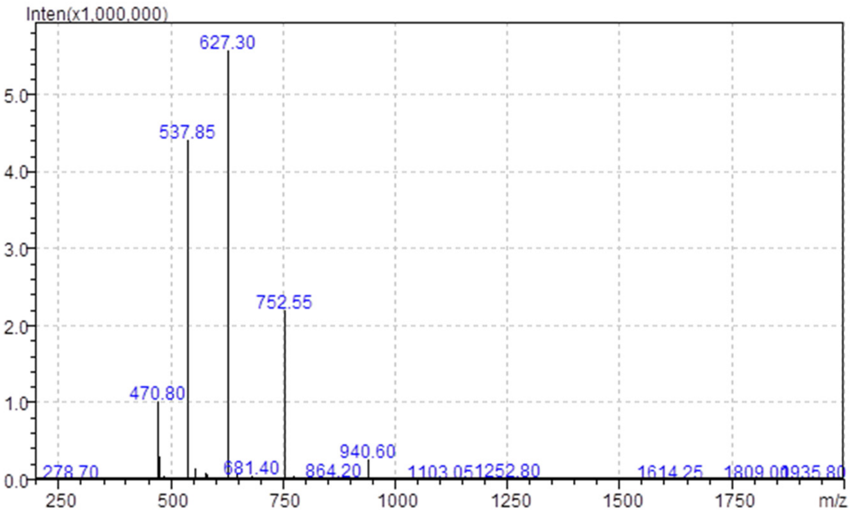

Positively charged ion series

Ch.	Average	MonoIso.
MH1+	3758.4016	3756.0193
MH2+	1879.7044	1878.5133
MH3+	1253.4720	1252.6780
MH4+	940.3559	939.7603
MH5+	752.4861	752.0097
MH6+	627.2397	626.8426
MH7+	537.7779	537.4376
MH8+	470.6816	470.3838
MH9+	418.4955	418.2308
MH10+	376.7467	376.5085

b) ECPep-D

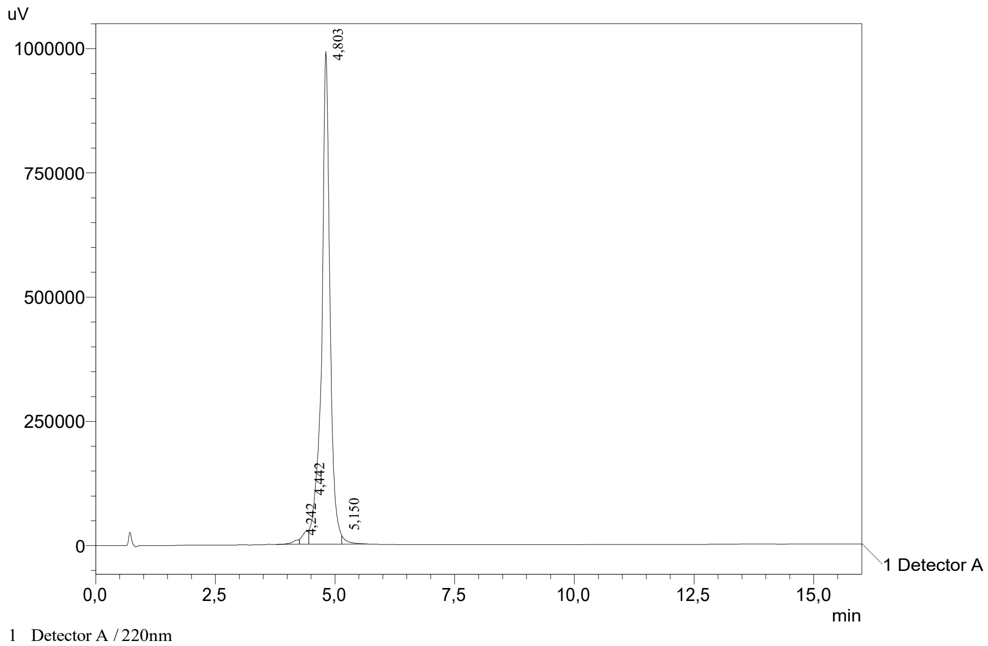

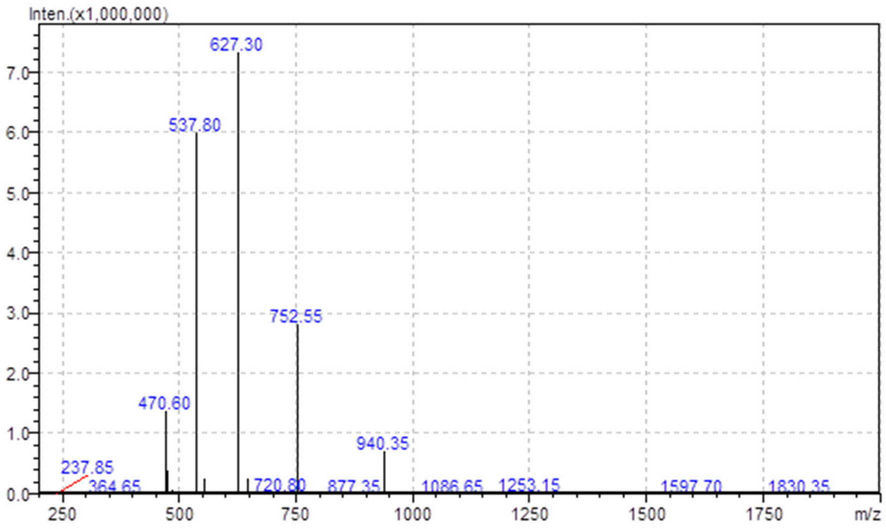

Positively charged ion series

Ch.	Average	MonoIso.
MH1+	3758.4016	3756.0193
MH2+	1879.7044	1878.5133
MH3+	1253.4720	1252.6780
MH4+	940.3559	939.7603
MH5+	752.4861	752.0097
MH6+	627.2397	626.8426
MH7+	537.7779	537.4376
MH8+	470.6816	470.3838
MH9+	418.4955	418.2308
MH10+	376.7467	376.5085

c) ECPep-2D-Orn

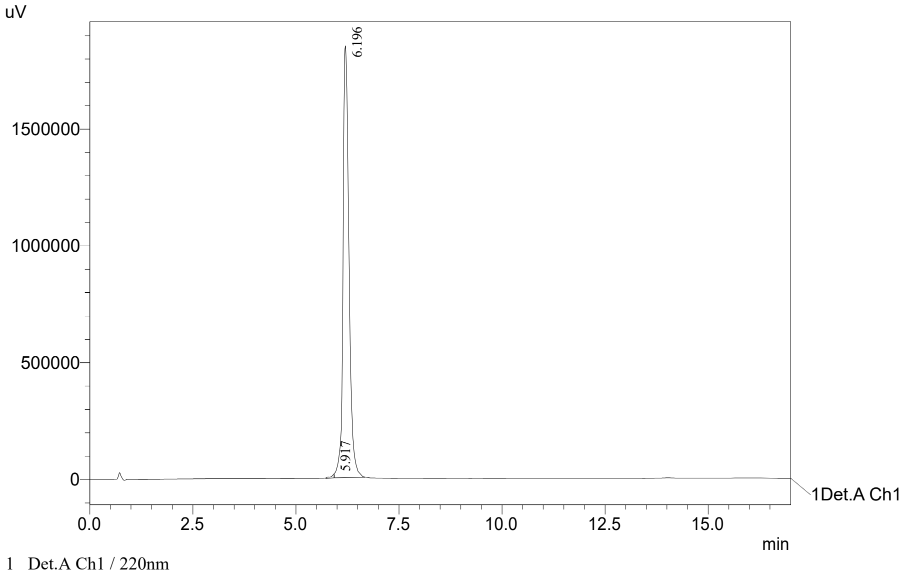

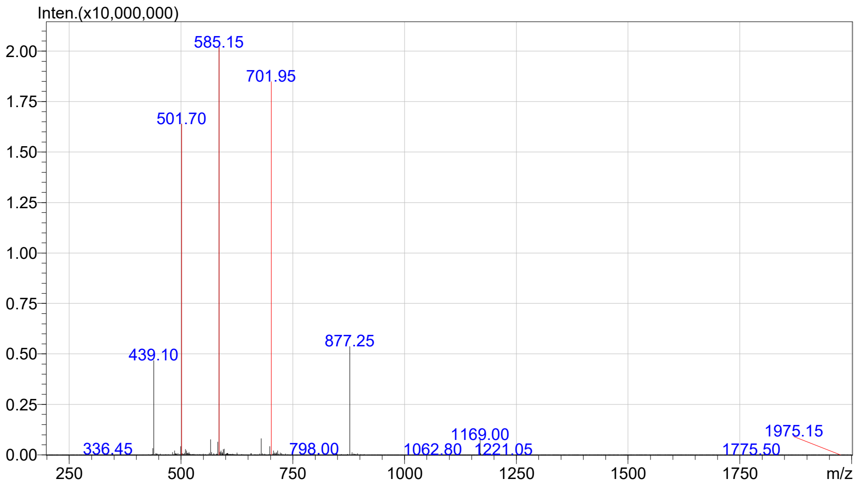

Positively charged ion series

Ch.	Average	MonoIso.
MH1+	3506.1599	3503.8885
MH2+	1753.5836	1752.4479
MH3+	1169.3915	1168.6344
MH4+	877.2954	876.7276
MH5+	702.0378	701.5835
MH6+	585.1994	584.8208
MH7+	501.7434	501.4189
MH8+	439.1514	438.8674
MH9+	390.4687	390.2163
MH10+	351.5225	351.2954

Figure S2. RP-HPLC and MS chromatograms of **a)** ECPep-L, **b)** ECPep-D and **c)** ECPep-2D-Orn. The Luna C_{18} (4.6 × 50 mm, 3 µm; Phenomenex) column was used to purify the peptides using the following gradient: buffer B (0.036% TFA in Acetonitrile) into buffer A (0.045% TFA in H_2O) over 15 min at 1 mL/min. Signal was registered at 220nm. The XBridge column C_{18} (4.6 × 150 mm, 3.5 µm, Waters) was then applied to check the MS of peptides with gradient buffer A (0.1% formic acid in water) and buffer B (0.08% formic acid in acetonitrile), 15 min at 1 mL/min, 220nm.

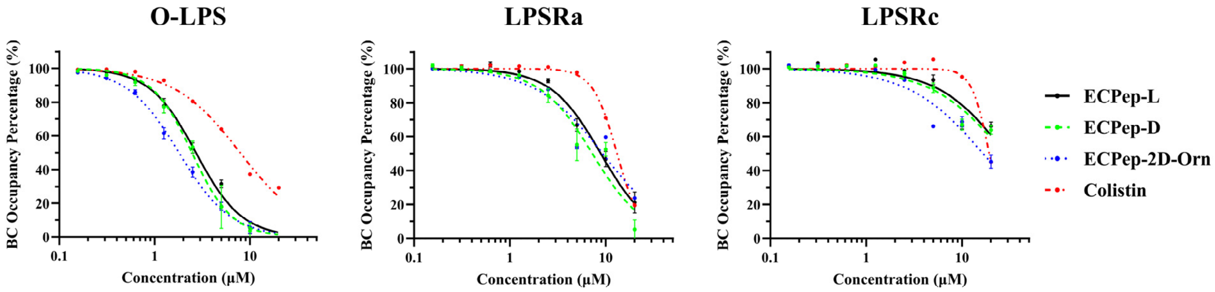

Figure S3. BODIPY-Cadaverine displacement curves for peptides and colistin. The curve was fitted by with nonlinear fit with normalized response.

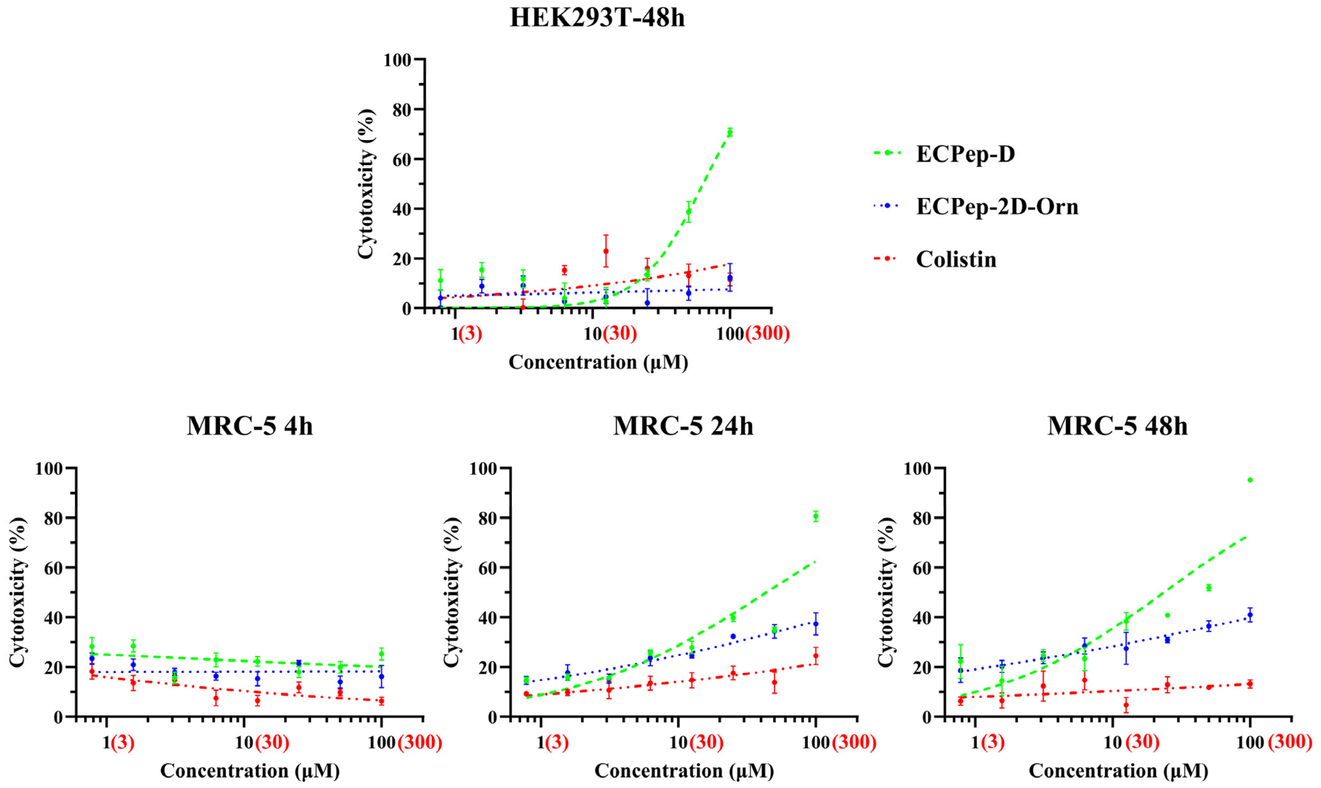

Figure S4. Cytotoxicity curves for peptides and colistin. The curve was fitted by with nonlinear fit with normalized response. The concentration of peptide was indicated in x-axis with black (0-100 µM) and that of colistin with red (0-300 µM).

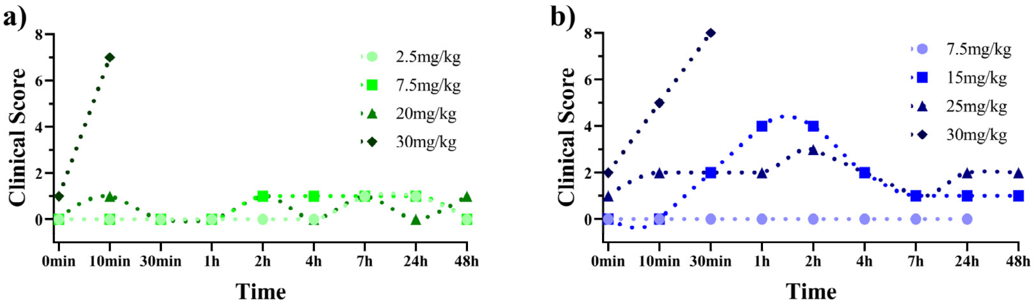

Figure S5. "Up and Down" assay. **a)** Clinical scores of ECPep-D at different concentration within 2 days. **b)** Clinical scores of ECPep-2D-Orn at different concentration within 2 days. Only 1 mouse in either group had been injected intraperitoneally from low dose to high dose and observed until 48h. The animal was euthanized when it had obvious clinical suffering.

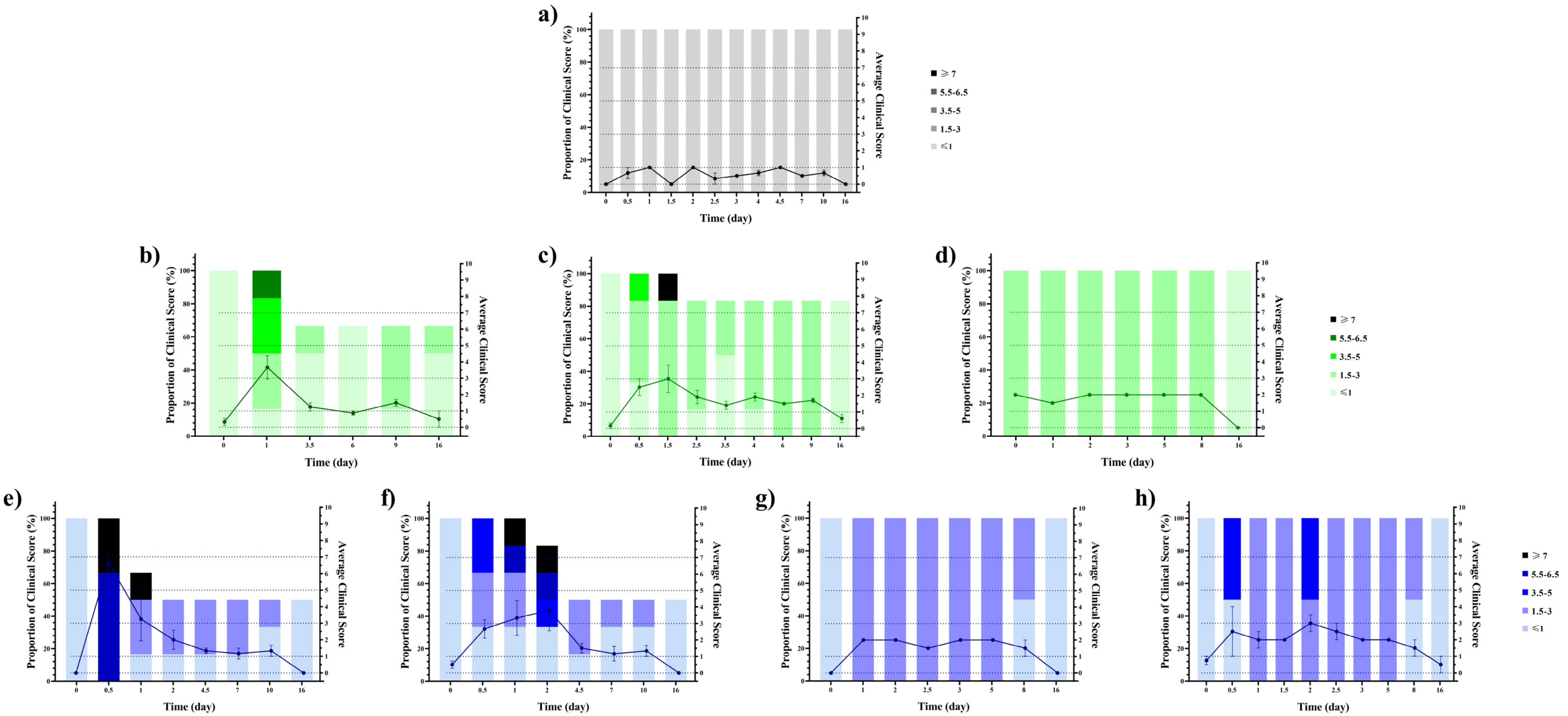

Figure S6. Changes of clinical score after each administration and survival percentages of animal for main toxicity assay. **a)** Clinical score at different days for Vehicle (HBS); **b-d)** Clinical score at different days for ECPep-D (b: 20 mg/kg, c: 15 mg/kg, d: 7.5 mg/kg); **e-h)** Clinical score at different days for ECPep-2D-Orn (e: 25 mg/kg, f: 20 mg/kg, g: 10 mg/kg, h: 7.5 mg/kg). In the first 3 days (from Day 0 to Day 2), which was the prescribed administration time, the clinical scores were recorded after each administration and from Day 3, when no administration were operated in any group, the clinical scores were only monitored at some specific days. The decrease of height of the column means euthanasia had been executed on the animal with high clinical score. Sample sizes and administration times in each group are indicated in Table S1.

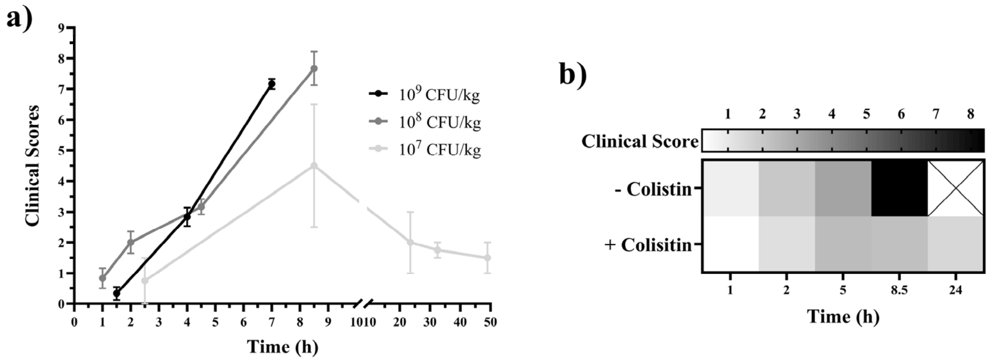

Figure S7. Clinical scores for the setting of murine acute infection model induced by *A. baumannii*. **a)** Time course change of mice clinical scores after incubation with different concentrations of *A. baumannii*. The treated groups were respectively 6 mice for 10^9 CFU/kg and 10^8 CFU/kg and 2 mice for 10^7 CFU/kg. **b)** Average clinical scores comparison of mice at different time points after incubation with 10^8 CFU/kg of *A. baumannii* in the absence or presence of colistin treatment. The treatment had been given at 2 h after bacteria administration. Four mice (2 male and 2 female) were used to compare between groups.

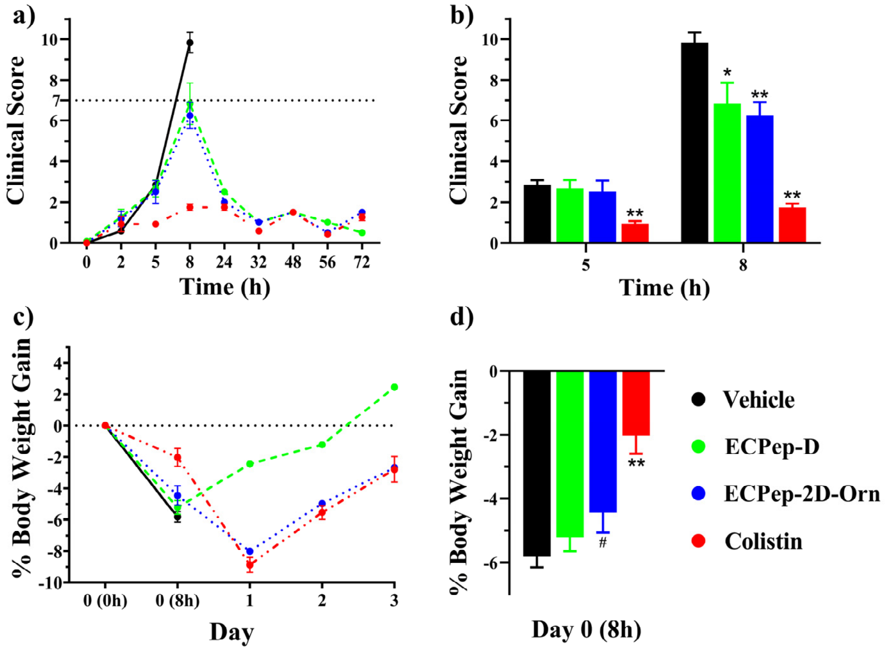

Figure S8. Changes clinical symptoms in 1st efficacy assays with 10 mg/kg peptides treatment. **a)** The average clinical score in 3 days; **b)** Histogram of clinical scores at 5 hours and 8 hours; **c)** The average body weight gain (%) at different hours in 3 days; **d)** Histogram of body weight gain (%) at 8 hours. Each animal had been inoculated 10^8 CFU/kg *A. baumannii* with 5% mucin before the first treatment. The different treatments were given 2 hours later after the bacteria inoculation respectively and last 3 days. Values are presented by mean ± SEM. Mann-Whitney test has been used for statistical comparison between different treatments and vehicle (***P* < 0.01, **P* < 0.05, $^{\#}$*P* < 0.1).

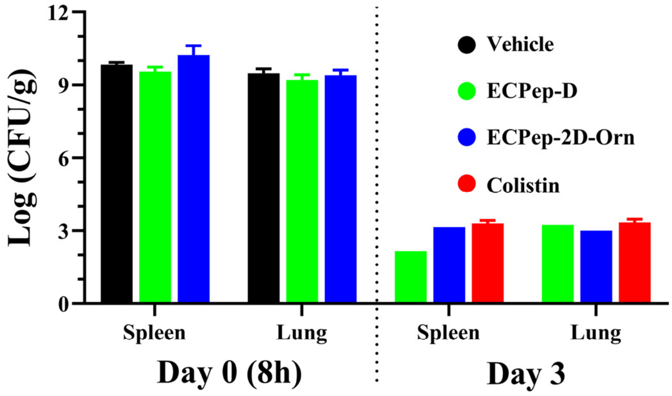

Figure S9. The average CFUs in organs (spleen and lung) of infected mice in the 1st efficacy assay with 10 mg/kg peptides treatment. Each animal had been inoculated 10^8 CFU/Kg *A. baumannii* with 5% mucin before treatment. Treatments were given 2 hours later after the bacteria inoculation respectively and last 3 days for the survival ones. Values are presented by mean ± SEM.

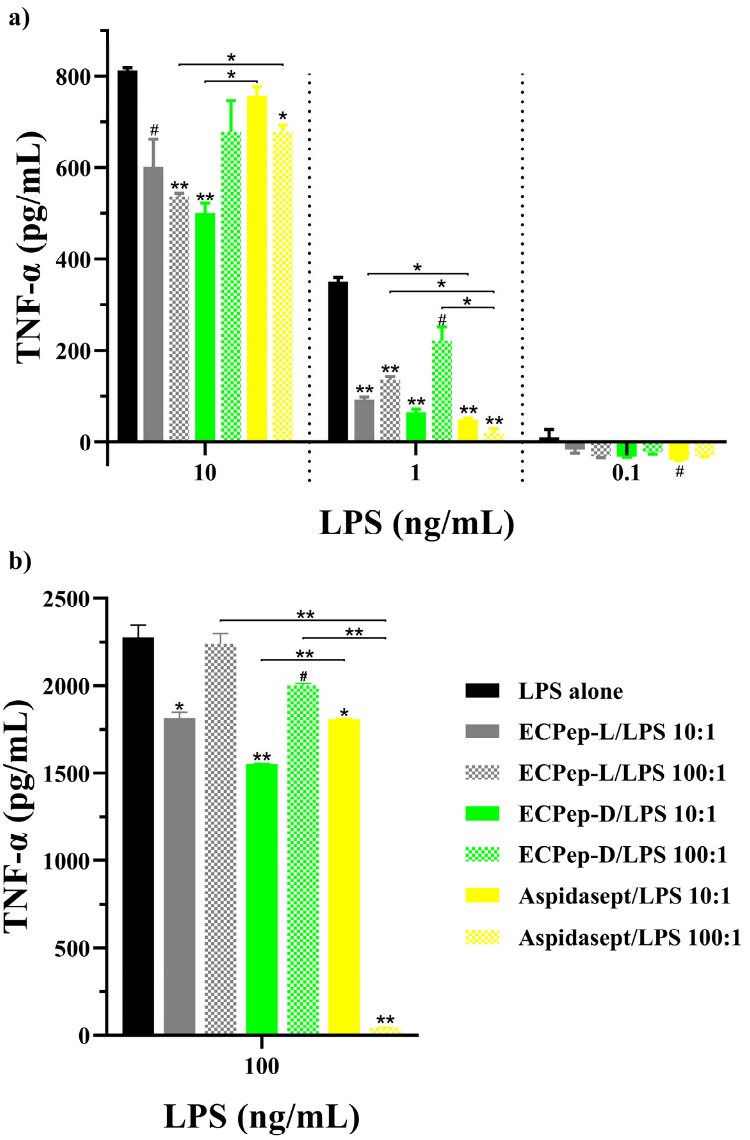

Figure S10. Inhibitory effect on LPS-induced TNF-*α* cytokine release. **a)** and **b)** Different concentration of LPS R60-induced secretion of TNF-a by human mononuclear cells and the inhibitory effect in the presence of the peptides ECPep-L, ECPep-D and Aspidasept ® was calculated. Unpaired t-test has been used for statistical comparison between peptides and Aspidasept ® within the same amount of LPS. Significant differences were compared with LPS alone sample when no group is indicated (**$P < 0.01$, *$P < 0.05$, #$P < 0.1$).
